# A risk-based framework to support conversion decision-making during minimally invasive left pancreatectomy: results from a multicentre registry

**DOI:** 10.1007/s13304-026-02632-8

**Published:** 2026-03-31

**Authors:** Salvatore Paiella, Matteo De Pastena, Erica Secchettin, Stefano Partelli, Niccolò Napoli, Giovanni Capretti, Sergio Alfieri, Giovanni Butturini, Alessandro Esposito, Massimo Falconi, Roberto Salvia, Alessandro Zerbi, Ugo Boggi, IGOMIPS Registry Study Group, Adelmo Antonucci, Gianandrea Baldazzi, Maria Luisa Boella, Felice Borghi, Umberto Bracale, Sergio Calamia, Donata Campra, Riccardo Casadei, Umberto Cillo, Davide Citterio, Andrea Coratti, Annalisa Comandatore, Raffaele Dalla Valle, Lorenzo De Franco, Fabrizio Di Benedetto, Greta Donisi, Giorgio Ercolani, Giuseppe Maria Ettorre, Giovanni Ferrari, Alessandro Ferrero, Marco Garatti, Gianluca Garulli, Alessandro Giardino, Antonio Giuliani, Salvatore Gruttadauria, Elio Jovine, Naomi Ingaglio, Angela Maffongelli, Giovanni Marchegiani, Marco Massani, Laura Mastrangelo, Riccardo Memeo, Carlo Molino, Luca Moraldi, Luca Morelli, Claudio Ricci, Alessia Ripolli, Renato Romagnoli, Raffaele Romito, Giovanni Domenico Tebala, Luca Tirloni, Leonardo Vincenti, Massimo Giuseppe Viola

**Affiliations:** 1 University of Verona, Verona, Italy; 2 Pancreatic Surgery Unit, Department of Surgery, Dentistry, Paediatrics and Gynaecology, University of Verona, Verona, Italy; 3 Pancreatic Surgery Unit, Department of Surgery, University of Verona Hospital Trust, Policlinico GB Rossi, Piazzale Scuro 10, 37134 Verona, Italy; 4 Department of Surgery, University of Verona Hospital Trust, University of Verona, Verona, Italy; 5 Pancreatic and Transplantation Surgical Unit, IRCCS San Raffaele Hospital, Vita-Salute University, Milan, Italy; 6 Division of General and Transplant Surgery, University of Pisa, Cisanello Hospital, Via Paradisa, 2, Pisa, Italy; 7 Department of Biomedical Sciences, Humanitas University, Pieve Emanuele, Milan Italy; 8 IRCCS Humanitas Research Hospital, Pancreatic Surgery Unit, Rozzano, Milan Italy; 9 Department of Medical and Surgical Sciences, Fondazione Policlinico Universitario A. Gemelli IRCCS, Rome, Italy; 10 Dipartimento Di Medicina E Chirurgia Traslazionale, Università Cattolica del Sacro Cuore, Rome, Italy; 11 Unit of HPB Surgery, Pederzoli Hospital, Peschiera del Garda, Verona, Italy; 12 Unit of Pancreatic Surgery, Department of Engineering for Innovation Medicine, Pancreas Institute, University of Verona, Verona, Italy; 13 UO Chirurgia Generale, Ospedale Sant’Anna, San Fermo Della Battaglia, Asst LarianaComo, Italy; 14 ASST Ovest Milanese, Nuovo Ospedale Di Legnano, Divisione Di Chirurgia Generale, Legnano, Italy; 15 Oncologic Surgery Unit Candiolo Cancer Institute, FPO-IRCCS, Candiolo, Turin, Italy; 16 Department of Gastroenterology, Endocrinology and Endoscopic Surgery, University Hospital of Naples, Federico II, Naples, Italy; 17 Department for the Treatment and Study of Abdominal Diseases and Abdominal Transplantation, Istituto Di Ricovero E Cura a Carattere Scientifico-Istituto Mediterraneo Per I Trapianti e Terapie Ad Alta Specializzazione (IRCCS ISMETT), University of Pittsburgh Medical Center Italy (UPMCI), Palermo, Italy; 18 Division of General and Emergency Surgery, Department of General and Specialist Medicine, Città Della Salute E Della Scienza University Hospital, Turin, Italy; 19 Division of Pancreatic Surgery, IRCCS, AOUBO, Bologna, Italy; 20 Department of Internal Medicine and Surgery (DIMEC), Alma Mater Studiorum, University of Bologna, Bologna, Italy; 21 HPB and Liver Transplantation Unit, Department of Surgical, Oncological and Gastroenterological Sciences, DISCOG, Padua University Hospital, Padua, Italy; 22 HPB Surgery and Liver Transplantation, Fondazione IRCCS Istituto Nazionale Tumori Di Milano, Milan, Italy; 23 Department of General Surgery, Emergency Surgery and Gastroenterological Diseases, Misericordia Hospital, Grosseto, Italy; 24 Division of General Surgery, University of Pisa, Pisa, Italy; 25 Department of Medicine and Surgery, University of Parma, Parma, Italy; 26 Hepato-Pancreato-Biliary Surgery and Liver Transplantation Unit, University Hospital of Modena, University of Modena and Reggio Emilia, Modena, Italy; 27 Department of Biomedical Sciences, Humanitas University, Pieve Emanuele, Milan, Italy; 28 General and Oncologic Surgery, Morgagni-Pierantoni Hospital, Forlì, Italy; 29 Department of General Surgery and Organ Transplantation, San Camillo Forlanini Hospital, Rome, Italy; 30 Division of Minimally Invasive Surgical Oncology, Niguarda Cancer Center, ASST Grande Ospedale Metropolitano Niguarda, Milan, Italy; 31 Department of General and Oncological Surgery, “Umberto I” Mauriziano Hospital, Turin, Italy; 32 Department of General Surgery, Fondazione Poliambulanza, Brescia, Italy; 33 Division of Surgery, Infermi Hospital, AUSL Romagna, Rimini, Italy; 34 General Surgery Unit, San Giuseppe Moscati Hospital, Aversa, Caserta Italy; 35 Department of General Surgery and Medical-Surgical Specialties, University of Catania, Catania, Italy; 36 Department of General Surgery, IRCCS Azienda Ospedaliero-Universitaria Di Bologna, Bologna, Italy; 37 Department of Oncological Surgery Team 1, “Antonio Cardarelli” Hospital, Naples, Italy; 38 General Surgery 1 Unit, Azienda ULSS 2 Marca Trevigiana, Treviso, Italy; 39 Department of Hepato-Biliary and Pancreatic Surgery, “F. Miulli” General Hospital, Acquaviva Delle Fonti, Bari, Italy; 40 Department of Medicine and Surgery, LUM University, Casamassima, Bari, Italy; 41 HPB Unit, Careggi University Hospital, Florence, Italy; 42 Division of General Surgery, Annalisa Comandatore, University of Pisa, Pisa, Italy; 43 General Surgery 2U-Liver Transplant Unit, Department of Surgery and Transplantation, Azienda Ospedaliero Universitaria Città Della Salute E Della Scienza Di Torino, Turin, Italy; 44 Azienda Ospedaliero Universitaria “Maggiore Della Carità”, Chirurgia Generale 2, Novara, Italy; 45 Department of Digestive and Emergency Surgery, Azienda Ospedaliera S.Maria, Terni, Italy; 46 Unit of Surgery, Department of Surgery Sciences, National Institute of Gastroenterology “S. de Bellis”, IRCCS Research Hospital, Castellana Grotte, Bari, Italy; 47 General Surgery Department, Ospedale Card. G. Panico, Tricase, Italy

**Keywords:** Left pancreatectomy, Minimally-invasive surgery, Robotic surgery, laparoscopy, Pancreatectomy

## Abstract

Minimally invasive left pancreatectomy (MILP) is increasingly adopted worldwide, but conversion to open surgery still occurs and is associated with poorer postoperative outcomes. Tools to identify patients at increased risk of conversion are limited. This multicentre registry-based study included patients undergoing laparoscopic (LLP) or robot-assisted (RLP) MILP within the IGOMIPS registry. Predictors of conversion were analysed using multivariable logistic regression. A simple preoperative risk score, the modified Conversion Risk Score (mCRS), was derived from independent predictors of conversion. We explored the interaction between preoperative risk, intraoperative complexity, and center-level variability. Of the 2127 MILPs included, 1235 (58.1%) were performed laparoscopically, and 892 (42.9%) were robot-assisted. Conversion occurred in 180 cases (8.5%) and was more frequent during LLP than RLP (11.8% vs 3.8%). Age ≥ 61 years, tumour diameter ≥ 35 mm, and preoperative suspicion of pancreatic malignancy were independently associated with conversion and formed the mCRS (range 0–5). Conversion rates increased progressively with higher mCRS values (ranging from 3.5% to 20.6%). When a high preoperative risk was combined with intraoperative complexity, the probability of conversion increased further (up to 19%). Differences in conversion rates between centres were primarily explained by case volume. In this large multicentre cohort, conversion during MILP was associated with patient age, tumour size, and suspected malignancy. The proposed mCRS, combined with intraoperative cues, may support risk stratification and timely intraoperative decision-making.

**Supplementary Information:**

The online version contains supplementary material available at https://doi.org/10.1007/s13304-026-02632-8.

## Introduction

Minimally invasive left pancreatectomy (MILP), including both robotic (RLP) and laparoscopic approaches (LLP), has become the preferred approach for body-tail tumors requiring surgery [1, 2]. Compared to traditional open surgery, MILP offers significant advantages, including reduced surgical trauma, shorter hospital stays, faster recovery, decreased postoperative pain, and improved cosmetic outcomes [3–5].

Despite these benefits, conversion to open surgery remains a relevant clinical issue, with reported rates varying widely from 3.5 to 30% [6–9]. Multiple patient-, disease-, and procedure-related factors and the surgical learning curve have been associated with conversion [10].

Two recent multicentre studies have explored conversion in depth by leveraging national and international registries, encompassing thousands of patients. In detail, De Ponthaud et al. analysed data from 2104 MILPs, including both RLPs and LLPs. They found a conversion rate of 6.5% for RLP and of 17% for LLP, and provided a score to predict conversion called “Conversion Risk Score” (CRS), which included variables such as male sex, BMI ≥ 25 kg/m^2^, history of laparotomy, tumor size ≥ 40 mm, initial pancreatitis, and planned splenectomy [6]. Müller et al., instead, investigated risk factors for conversion in a large population of 2452 patients who underwent RLP. They reported an overall conversion rate of 3.1%, with risk factors for conversion including tumor size greater than 51 mm, previous abdominal surgery, patients outside benchmark criteria, and age greater than 62 years [11]. In both studies, conversion was associated with worse postoperative outcomes, including failure to achieve textbook outcomes [6, 11], underscoring the importance of preventing or early planning conversion.

This study sought to identify and quantify predictors of conversion in a large, multicentre cohort that included RLP and LLP. It also aims to develop a novel conversion risk score, assess the impact of conversion on postoperative outcomes, and refine risk-stratification tools for informed clinical decision-making.

## Methods

### IGOMIPS registry

The IGOMIPS registry and its methodology have been previously described in detail [12], and it has already been used to provide a snapshot of MILP use in Italy [13]. The registry includes, among others, surgical and oncological data of patients who underwent minimally invasive pancreatic surgery at participating centres. It was established and approved by the Independent Ethics Committee of the Humanitas Institute (authorization number 2167) in 2019. IGOMIPS was recorded in the Registry of Patient Registries (RoPR) of the Agency for Healthcare Research and Quality, US Department of Health (Registry of Patient Registries. Content last reviewed November 2025  https://www.ahrq.gov/ropr/index.html). Each center received institutional approval to join IGOMIPS and to collect data. The IGOMIPS registry develops under the umbrella of the Italian Association for the Study of the Pancreas (AISP) and has been endorsed by the following national surgical societies: Italian Association of Hepatobiliopancratic Surgery (AICEP), Italian Society of Surgery (SIC), Italian Society of Surgical Endoscopy (SICE), and Association of Italian Hospital Surgeons (ACOI). The study was conducted according to the Helsinki Declaration ethical principles, and reporting followed the Strengthening the Reporting of Observational Studies in Epidemiology (STROBE) guidelines [14].

### Population

The IGOMIPS steering committee evaluated and approved the study proposal, after which a call for participation was issued to all participating centres. Among the 45 centres joining the registry, 43 accepted to participate and contributed to the final analytic cohort. On 30th November 2025, the registry was queried retrospectively for MILP cases performed from September 2019 to May 2023. Adult patients (≥ 18 years) who underwent MILP were included, regardless of indication. Patients with incomplete clinical or pathological data, a previous history of pancreatectomy, or cases involving hybrid, hand-assisted procedures, or initially open procedures were excluded.

### Data collection

Demographic features (age, sex, BMI, comorbidities, previous abdominal open surgery), tumor characteristics (size, location, benign vs. malignant condition, any preoperative oncological treatment for malignancies), surgical variables (RLP or LLP, operative time, intraoperative blood loss, centre’s annual caseload, and postoperative data (major complications, defined as ≥ 3 per Clavien-Dindo classification[15], pancreatic surgery-specific complications [16–18], length of stay, ICU admission, readmissions, reoperation rate, conversion rate and mortality.

### Study endpoints

The study’s primary endpoint was to assess MILP conversion rates and investigate predictive risk factors for conversion to open. Secondary endpoints included: a comparison of postoperative outcomes between converted and non-converted cases; creation of a novel CRS, called modified CRS (mCRS) [6]; development of a “conversion point” [19, 20] to support early, safe intraoperative decision-making; and a center-specific analysis to explore whether the volume of cases impacted the conversion rate. Centres were stratified into low-, medium-, and high-volume groups according to tertiles of total MILP caseload within the study cohort.

### Statistical analysis

Continuous variables were summarized as mean ± standard deviation and compared between converted and non-converted patients using the Mann–Whitney U test. Categorical variables were reported as counts and percentages and compared using the χ^2^ test.

Variables associated with conversion at univariate analysis (p < 0.10) and those deemed clinically relevant based on prior literature were entered into multivariable logistic regression models to identify independent predictors of conversion to open surgery. Results were reported as odds ratios (ORs) with corresponding 95% confidence intervals (CIs). Multicollinearity among covariates included in multivariable models was assessed using variance inflation factors (VIF), and no evidence of problematic collinearity was identified (all VIF values were below 2, indicating minimal multicollinearity). Model discrimination was evaluated using the area under the receiver operating characteristic (ROC) curve. Two-sided p-values < 0.05 were considered statistically significant. Statistical analyses were performed using R (v. 3.2.2; R Foundation for Statistical Computing, Vienna, Austria).

### Development of a modified conversion risk score (mCRS)

Variables included in the mCRS were selected a priori based on three criteria: independent association with conversion in multivariable analysis, availability at the time of surgical planning, and clinical interpretability. Although additional variables were explored in extended models, only age, tumor size, and preoperative suspicion of pancreatic malignancy demonstrated robust and consistent independent effects and were therefore retained in the final score to preserve parsimony and generalizability. Descriptive variables and postoperative outcomes were excluded because they cannot serve as predictors of intraoperative conversion. This approach limits overfitting and preserve preoperative applicability. According to univariate analysis (**Supplementary Table S2**) and prior literature [6, 8, 11], the variables included in the model were male sex, BMI, and the presence of prior laparotomy (defined as any open procedure, excluding caesarean surgery, appendectomy, or uro-gynecological procedures that did not involve abdominal components).

BMI was further explored as a potential predictor of conversion using ROC-derived thresholds. However, it was not included in the model to preserve its simplicity and interpretability, as it resulted in a modest improvement in model discrimination (+ 0.007 increase in the AUC) without a clinically meaningful cutoff (25.9 kg/m^2^). To avoid arbitrary cutoffs derived from external literature, all other continuous predictors were dichotomized using ROC-based thresholds (maximum Youden index). The following cutoffs were identified within this cohort: age ≥ 61 years and tumor diameter ≥ 35 mm. Preoperative histology was categorized into two groups based on the best-performing regression model: any pancreatic malignancies versus all other diagnoses. Coefficients (β) from the logistic regression were then transformed into a simple additive score by dividing each coefficient by the smallest significant coefficient and rounding to the nearest integer.

The final mCRS point assignment was:

- Tumor ≥ 35 mm →  + 1 point
- Age ≥ 61 years →  + 2 points
- Preoperative suspicion of pancreatic malignancy →  + 2

The resulting mCRS ranges from 0 to 5 points. Model discrimination was evaluated using ROC analysis (AUC). Calibration was assessed descriptively by observed conversion rates across mCRS strata.

Additionally, to investigate whether the surgical approach (laparoscopic vs robot-assisted) influenced the relationship between preoperative risk and conversion, a subgroup analysis comparing LLP and RLP was performed. Patients were stratified into three risk classes based on their mCRS: low-risk (0–1), intermediate-risk (2–3), and high-risk (4–5). The impact of the surgical approach was assessed by including an interaction term in the multivariable logistic regression model, as presented in the Supplementary Material. A p-value for < 0.05 was considered statistically significant, indicating that the impact of mCRS on conversion probability differed between the two techniques.

### Conversion point analysis

To build an intraoperative decision-support framework for identifying an elective potential conversion point, the preoperative mCRS was combined with dynamic intraoperative parameters, including operative time exceeding 300 min and estimated blood loss (EBL) exceeding 300 mL. The operative time and EBL thresholds were chosen using sensitivity analyses and literature data.

Two risk tiers and four groups were identified: an increased-risk zone (mCRS ≥ 4) and a high-risk conversion-point zone (mCRS ≥ 4 plus intraoperative factors, such as time > 300 min and EBL > 300 mL).

### Center-effect analysis

Center-level analyses were performed to contextualize inter-institutional variability in conversion rates rather than to estimate a causal effect of institutional performance. For each participating center, the conversion rate and corresponding 95% confidence intervals were calculated using Wilson’s method. Center-specific conversion rates were displayed in forest plots. In contrast, funnel plots were constructed to assess whether the observed variability exceeded what would be expected based on case volume and random variation. Center procedural volume was defined as the total number of MILP procedures contributed to the analytic cohort. Centres were stratified into tertiles of procedural volume (low-, medium-, and high-volume) to explore volume-related trends, and the Cochran–Armitage test was used to assess linear trends in conversion rates across volume categories. To account for differences in case-mix across centres, center-level analyses were complemented by risk-adjusted approaches. Expected probabilities of conversion were derived from the multivariable model and aggregated at the center level to compare observed versus expected conversion rates. In addition, center-specific conversion rates were explored within predefined mCRS risk strata to assess whether apparent inter-center variability persisted after stratification by preoperative risk. Multivariable modelling with center as a fixed effect was not pursued due to the large number of centres and sparse outcomes across several institutions, which would have led to unstable, overparameterized estimates.

### Missing data handling

No imputation of missing values was performed, as sensitivity analyses did not reveal relevant differences in baseline characteristics between patients with complete and incomplete data for the variables included in the multivariable model.

## Results

A total of 2,127 patients met the inclusion criteria. 1,771 (83.2%) were planned LP with splenectomy, and 356 (16.8%) were planned LP with splenic preservation. LLP and RLP were performed in 1,235 (58.1%) and 892 (42.9%) cases, respectively. Conversion occurred more frequently during LP with splenectomy, particularly in laparoscopic procedures (13.1%), compared with the robot-assisted approach (4.4%). Spleen-preserving procedures were associated with lower conversion rates, in both in laparoscopic (3.1%) and robotic (1.6%) approaches. A discrepancy was observed between the planned and performed procedures in 84 cases (3.9%). Among these cases, 69 planned spleen-preserving procedures required splenectomy, and 15 planned pancreatectomies with splenectomy were completed with splenic preservation. A gradual reduction in conversion rate was observed over time, from 10.4% in 2019 to 6.3% in 2023, although this trend did not reach statistical significance (Cochran–Armitage test for trend, p = 0.118, Figure S1). Preoperative complexity differed between LLP and RLP in the overall cohort. Laparoscopic cases included a higher proportion of high-risk patients (mCRS 4–5), whereas robotic procedures were not selectively limited to low-risk profiles (Supplementary Table S1).

### Baseline characteristics

Among the baseline and operative variables analysed, several factors showed statistically significant associations with conversion (Table 1). In converted patients, age and BMI were significantly higher (69 [IQR 61–76] vs 64 [53–73] years, p < 0.001, and 25.9 vs 24.8 kg/m^2^, p = 0.004, respectively). Converted procedures demonstrated longer operative time (295 vs 245 min; p < 0.001) and greater estimated blood loss (200 vs 100 mL, p < 0.001). In converted cases, the tumor size was bigger (31 vs 27 mm, p = 0.012), and the preoperative suspicion of pancreatic malignancy was more frequent (56.1% vs 28, p < 0.001).

**Table 1:** Baseline and operative characteristics according to conversion

Variable	No conversion (n = 1947)	Conversion (n = 180)	p-value
Age (years)^*^	64 [53–73]	69 [61–76]	** < 0.001**
BMI, kg/m^2*^	24.8 (22.3–27.8)	25.9 (23.2–29.3)	**0.004**
Sex,			0.059
F	1065 (54.7)	82 (45.6)	
M	881 (45.2)	98 (54.4)	
ASA			0.077
I–II	1277 (65.6)	103 (57.2)	
III-IV	659 (33.8)	76 (42.2)	
Estimated blood loss^*^ (ml)	100 (50–200)	200 (150–500)	** < 0.001**
Operative time (min)^*^	245 (199–310)	295 (240–360)	** < 0.001**
Tumor size (mm)^*^	27 (19–40)	31 (20–46.5)	**0.012**
Prior abdominal open surgery^§^	532 (27.9)	61 (33.9)	0.073
Final histology			** < 0.001**
Pancreatic malignancy ^‡^	553 (28.4)	101 (56.1)	
Neuroendocrine tumor	558 (28.7)	25 (13.9)	
Cystic tumor	480 (24.7)	20 (16.7)	
Other	356 (18.3)	24 (13.3)	

Values in parentheses are percentages unless indicated otherwise; values are ^*^median with interquartile range

Values in bold are statistically significant

ASA: American Society of Anesthesiologists; BMI: body mass index

^§^Appendectomy, caesarean and urogynecological procedures that did not involve abdominal surgical phases or interventions on abdominal organs were excluded

^‡^Including the following histologies: pancreatic ductal adenocarcinoma, Intraductal Papillary Mucinous Neoplasm-carcinoma, and neuroendocrine carcinoma

### Multivariable predictors of conversion

In the adjusted multivariable model, three variables independently predicted conversion (Table 2): age ≥ 61 years (OR 2.1, 95%CI [1.38 – 3.21], p < 0.001), tumor size ≥ 35 mm (OR 1.52, 95%CI [1.03–2.25], p = 0.036, and pancreatic malignancy (OR 1.97, 95%CI [1.35–2.88], p < 0.001). Male sex, a BMI of ≥ 25, and a prior laparotomy were not independently predictive.

**Table 2:** Adjusted multivariable analysis including additional covariates (this model was not used for score derivation)

Variable	β (coef)	OR (95% CI)	p-value
Age ≥ 61 years	0.74	2.1 (1.38–3.21)	** < 0.001**
Tumor diameter ≥ 35 mm	0.42	1.52 (1.03–2.25)	**0.036**
Pancreatic malignancy vs other histotypes	0.68	1.97 (1.35–2.88)	** < 0.001**
Male sex	0.11	1.12 (0.82–1.54)	0.460
BMI ≥ 25 kg/m^2^	0.17	1.19 (0.86–1.64)	0.290
History of laparotomy	0.20	1.22 (0.87–1.71)	0.250

Values in bold are statistically significant

BMI: body mass index

### Performance of the mCRS

**Fig. 1:**
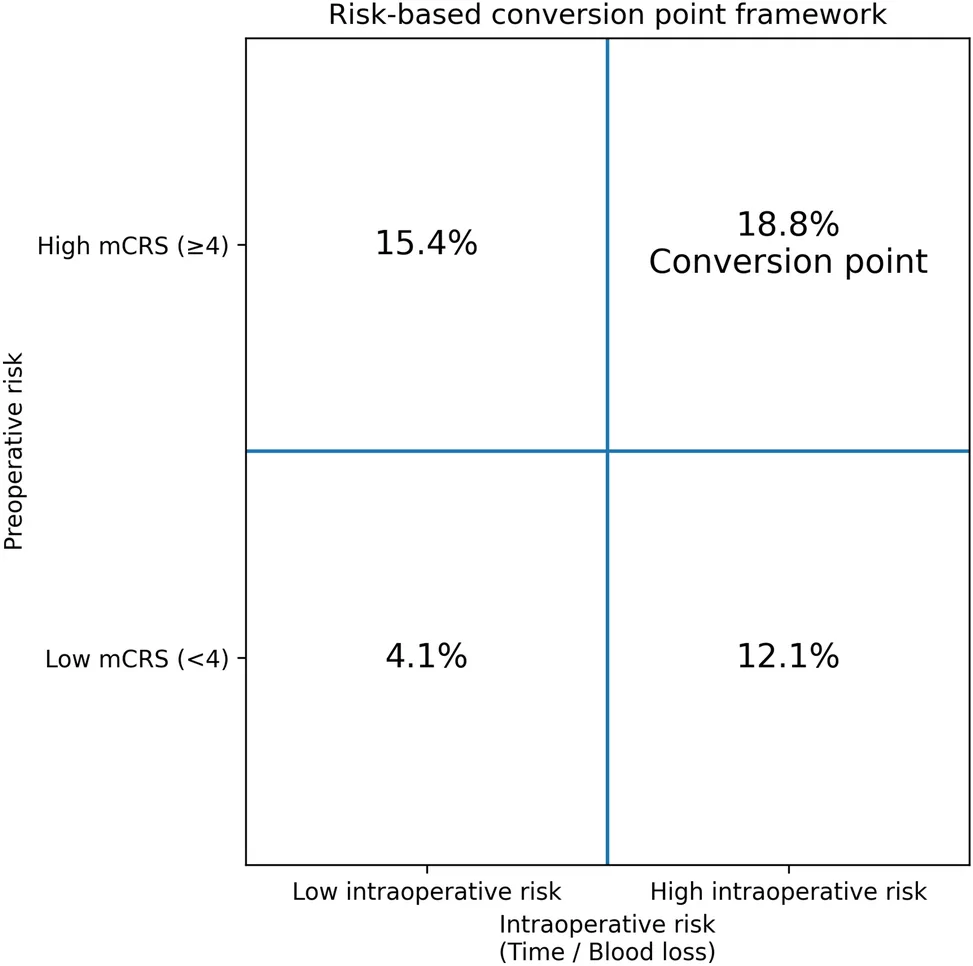
Combined preoperative and intraoperative risk stratification

The mCRS demonstrated monotonically increasing conversion risk (**Figure S2**), ranging from 3.5% (score 0) to 20.6% (score 5). It exhibited moderate discrimination (AUC 0.663, **Figure S3**), similar to the full multivariable model, confirming good internal validity for a simplified preoperative score. When preoperative mCRS was combined with intraoperative risk factors, conversion risk increased substantially (Table 3). Patients with mCRS ≥ 4 already carry a substantially increased risk of conversion (> 13%, so-called “high-risk group”), even in the absence of significant intraoperative stress. When a high mCRS was combined with intraoperative complexity (operative time > 300 min and/or EBL > 300 mL), the observed conversion rate approached 19%, defining a “conversion point” at which elective conversion may be preferable to continued minimally invasive dissection (Fig. 1).

**Table 3:** Conversion rate stratified per modified Conversion Risk Score (mCRS) and approach

mCRS-based risk of conversion	LLP (n = 1,235)	RLP (n = 892)	p-value
Low (Score 0–1)	6.4% (28/282)	2% (5/256)	**0.020**
Medium (Score 2–3)	13.6% (42/309)	4.3% (12/282)	** < 0.001**
High (Score 4–5)	21.5% (49/228)	6.1% (9/147)	** < 0.001**

Values in bold are statistically significant

mCRS: modified Conversion Risk Score; LLP: laparoscopic left pancreatectomy; RLP: robot-assisted left pancreatectomy

The predictive value of mCRS remained consistent across the three conversion risk categories (Table 3). However, the absolute risk of conversion was markedly lower in the robotic cohort across all risk strata. In the high-risk group (mCRS 4–5), the conversion rate was 21.5% (49/228) for LLP compared to 6.1% (9/147) for RLP (p < 0.001, **Figure S4**). Each one-point increase in mCRS was associated with a higher risk of conversion in both LLP (OR 1.45) and RLP (OR 1.41); however, the interaction between mCRS and surgical approach was not statistically significant (p for interaction = 0.799), indicating a similar relative effect of mCRS across surgical approaches.

Sensitivity analyses using alternative operative time thresholds (240 and 360 min) yielded consistent results, with progressively increasing conversion rates across thresholds (**Supplementary Table S3**).

### Postoperative Outcomes

Postoperative outcomes are listed in Table 4. Converted patients had a significantly longer postoperative length of stay compared with non-converted patients (median 8 [IQR 6–13] vs 7 [5–9] days, p < 0.001). This difference remained significant when analyses were stratified by surgical approach, both in laparoscopic (p < 0.001) and robotic (p = 0.022) procedures. Furthermore, they experienced higher rates of severe in-hospital complications (Clavien–Dindo ≥ III, 16.5% vs 6.1%, p < 0.001), increased reintervention rate (10.3% vs 2.9%, p < 0.001), postoperative pancreatic fistula (32.7% vs. 22.4%, p = 0.007), delayed gastric emptying (8.8% vs. 1.3%, p < 0.001), post-pancreatectomy haemorrhage (8% vs 3.6%, p = 0.009), sepsis (12.5% vs 4.1%, p < 0.001), any medical and surgical complications (p < 0.05), and 90-day mortality (3.6% vs. 0.8%, p = 0.005). In multivariable analysis adjusted for age and surgical approach, conversion was not independently associated with major complications at 90 days (OR 1.56, 95% CI 0.93–2.62, p = 0.093).

**Table 4:** Postoperative outcomes by conversion

Outcome	No conversion (n = 1947)	Conversion (n = 180)	p-value
Length of stay (overall, days)^*^	7 (5–9)	8 (6–13)	** < 0.001**
LLP	7 (5–9)	8 (6–13)	** < 0.001**
RLP	7 (5–9)	8 (7–12)	**0.022**
In-hospital major complications (CD ≥ III)	115 (6.1)	29 (16.5)	** < 0.001**
90-day major complications (CD ≥ III)	136 (7.6)	19 (11.4)	0.103
90-day mortality	14 (0.8)	6 (3.6)	**0.005**
Reintervention	54 (2.9)	18 (10.3)	** < 0.001**
Readmission	227 (12.2)	28 (16.3)	0.154
Any surgical complication	577 (30.4)	67 (38.1)	**0.044**
Any medical complication	331 (17.5)	52 (29.5)	** < 0.001**
POPF	330 (22.4)	48 (32.7)	**0.007**
SSI	21 (1.1)	5 (2.8)	0.106
Chyle leak	35 (1.9)	7 (4)	0.103
DGE	15 (1.3)	9 (8.8)	** < 0.001**
PPH	69 (3.6)	14 (8)	**0.009**
POFC	344 (18.2)	38 (21.6)	0.313
Sepsis	77 (4.1)	22 (12.5)	** < 0.001**

Values in parentheses are percentages unless indicated otherwise; values are ^*^median with interquartile range

Values in bold are statistically significant

LLP: laparoscopic left pancreatectomy; RLP: robot-assisted left pancreatectomy; CD: Clavien-Dindo classification; POPF: postoperative pancreatic fistula (ISGPS grades B and C); SSI: surgical site infection (any); DGE: delayed gastric emptying (any grade); PPH: post-pancreatectomy hemorrhage (any grade); POFC: postoperative fluid collections (defined as any postoperative fluid collection that needed to be treated)

### Center-effect analysis

A total of 43 centres contributed patients to the final analytic cohort. The number of MILPs performed per institution varied widely (range, 1–251 cases). Conversion rates across centres demonstrated substantial heterogeneity, ranging from 0 to 50% (**Figure S5**), although centres with the highest or lowest rates often had very small case volumes. The conversion rate increased as the surgical volume decreased, with high-, medium-, and low-volume centres reporting conversion rates of 8.2%, 8.6%, and 11.8%, respectively (Cochran-Armitage test for trend, p = 0.3). The funnel plot analysis demonstrated that most centres, including high-volume institutions, fell within the expected 95% and 99.8% control limits (**Figure S6**), suggesting that the observed heterogeneity is mainly attributable to case volume and random variation rather than systematic center-specific effects. Risk-adjusted (for patient-level risk factors such as age ≥ 61 years, tumor diameter ≥ 35 mm, and preoperative suspicion of pancreatic malignancy) funnel plot showed that most centres clustered around the expected conversion rate, with no evidence of systematic deviation (**Figure S6**).

## Discussion

We analysed preoperative and intraoperative determinants of conversion during MILP. We confirm [6, 11, 21] that conversion is an uncommon but clinically relevant event, affecting approximately 8.5% of cases and occurring more frequently during LLP (11.8%) than during RLP (3.8%). A dedicated comparison between laparoscopic and robot-assisted approaches regarding spleen-preserving distal pancreatectomy using IGOMIPS data has shown similar conversion rates, whether considering unmatched or propensity score-matched cohorts. In that series, pancreatic malignancy histology was an independent predictor of conversion to surgery [22].

Converted procedures were technically more demanding, as reflected by longer operative times and greater blood loss. Although converted patients had worse postoperative outcomes, conversion was not independently associated with major complications after adjustment (OR 1.56, 95%CI [0.93–2.62], p = 0.093).

Several scores have been proposed to predict conversion [6, 23]. However, unlike previously proposed models, our approach integrates a simple preoperative score with intraoperative parameters to support real-time decision-making. The mCRS developed integrates three universally available preoperative factors, such as age ≥ 61 years, tumor diameter ≥ 35 mm, and preoperative suspicion of pancreatic malignancy. Although its discriminative ability was moderate (AUC 0.663), the mCRS demonstrated a monotonically increasing conversion risk. For high-risk cases (mCRS ≥ 4), which already carry a substantially increased baseline risk of > 13%, given the worse postoperative outcome, the score may be helpful as a preoperative stratification tool (e.g., using the robot-assisted approach or choosing an open approach, when the minimally invasive expertise may be insufficient).

The mCRS proposed might be considered a surrogate tool for evaluating MILP difficulty, and unlike MILP difficulty scores [24, 25], it comprises only three variables which are easy to obtain, always available at the time of surgery, and which have already been included in the other conversion risk scores [6, 23], demonstrating that they are core determinants of intraoperative conversion to open surgery. Crucially, we also introduce an intraoperative decision-support algorithm, the “MILP conversion point” framework, which combines the mCRS with dynamic intraoperative factors (operative time > 300 min and EBL > 300 mL). Our proposed framework stratifies risk defining two risk-zones: a low-risk zone, when the mCRS is < 4 and there are low intraoperative risk factors (time ≤ 300 min and EBL ≤ 300 mL), where the conversion rate is 4.1%, and a high-risk zone, when the mCRS is ≥ 4 and is combined with high intraoperative risk factors, such as operative time > 300 min or EBL > 300 mL, where the conversion rate approaches 19%.

This framework provides a practical tool for identifying high-risk scenarios and supporting timely intraoperative decisions, thereby potentially avoiding the adverse outcomes associated with delayed or urgent conversions [10].

Although interaction analysis did not demonstrate a statistically significant effect modification, RLP was associated with consistently lower absolute conversion rates across increasing mCRS categories. In high-risk patients (mCRS 4–5), RLP had a markedly lower conversion rate compared with laparoscopy (6.1% vs 21.5%, p < 0.05), although causality cannot be inferred. This may be likely due to the benefits of the robotic platform, such as enhanced dexterity, stable three-dimensional visualization, and improved ergonomics.

Unlike some reports, which identified BMI as an independent predictor of conversion [6, 8, 9, 21], BMI did not reach statistical significance in our multivariable model once tumor size and preoperative suspicion of malignancy were accounted for, suggesting that body habitus alone is insufficient to explain the technical difficulty of MILP when other factors predominate.

Because conversion is often precipitated by cumulative intraoperative difficulty, an exploratory “conversion-point” analysis was performed by integrating the mCRS with intraoperative risk factors for conversion, such as operative time and EBL. As such, we identified an inflection point at which elective conversion may be safer than continued minimally invasive dissection. This framework parallels safety-based decision algorithms described in other surgical fields and may help avoid the adverse outcomes associated with delayed urgent conversion.

Center-level analysis revealed apparent variability in conversion rates, primarily driven by differences in case volume. There was a numerical trend towards higher conversion rates in low-volume centres (11.8%) than with medium- (8.6%) and high-volume centres (8.2%), although this difference did not reach statistical significance (p = 0.3). The funnel plot showed that most centres clustered within the expected control limits, with only a minority of small-volume centres exhibiting borderline outlying behaviour. This pattern persisted after adjustment for patient-level risk factors for conversion (age, tumor size, and preoperative suspicion of pancreatic malignancy). Although individual surgeon learning curves could not be directly evaluated within the registry, institutional procedural volume reasonably serves as a surrogate of cumulative expertise. Surgical volume likely reflects cumulative institutional experience, influencing conversion risk through case selection and intraoperative judgment. Accordingly, an independent center effect was not identified. This study was not designed to re-demonstrate the well-established relationship between surgical experience and outcomes, but rather to contextualize conversion risk within a risk-adjusted, multicentre framework [10].

This study is limited by its retrospective design, case-mixing, and incomplete availability of certain technical variables, such as the type of conversion to open (planned vs. urgent), which was available for only a minority of cases. Furthermore, the registry does not systematically record segmental tumor location (proximal body versus tail) and the proximity of the tumor to peripancreatic vessels (e.g., splenoportomesenteric confluence), which may represent an additional determinant of technical difficulty and conversion risk. Additionally, the AUC of the mCRS was modest, and its external validation in independent cohorts is warranted.

In conclusion, this multicentre analysis identifies age, tumor size, and preoperative suspicion of pancreatic malignancy as key determinants of conversion during MILP and introduces a practical risk-based “conversion point” concept that may support safer intraoperative decision-making. Standardized risk-based decision pathways may help reduce inter-center variability and improve outcomes in MILP.

## Supplementary Information

Below is the link to the electronic supplementary material.

Supplementary file1 (DOCX 1116 KB)

## Data Availability

The dataset generated during the current study are available from the corresponding author on reasonable request. All materials are available upon request.
