# Supplementary material for: A risk-based framework to support conversion decision-making during minimally invasive left pancreatectomy: results from a multicentre registry
Source: Updates Surg. 2026 Mar 31;78(7):2853–62. doi: 10.1007/s13304-026-02632-8 (PMC13751565; doi:10.1007/s13304-026-02632-8)
Supplement: Supplementary file 1 [file 13304_2026_2632_MOESM1_ESM.docx]

**A Risk-Based Framework to Support Conversion Decision-Making During Minimally Invasive Distal Pancreatectomy: Results from a Multicentre Registry**

*Running title*: Conversion risk in minimally invasive left pancreatectomy

Salvatore Paiella^*1,2^, Matteo De Pastena^*3^, Erica Secchettin^4^, Stefano Partelli^5^, Niccolò Napoli^6^, Giovanni Capretti^7,8^, Sergio Alfieri^9,10^, Giovanni Butturini^11^, Alessandro Esposito^3^, Massimo Falconi^5^, Roberto Salvia^1, 12^, Alessandro Zerbi^7,8^, Ugo Boggi^6^, & IGOMIPS Registry Study Group

^1^ University of Verona, Verona, Italy

^2^ Pancreatic Surgery Unit, Department of Surgery, Dentistry, Paediatrics and Gynaecology, University of Verona, Verona, Italy

^3^ Pancreatic Surgery Unit, Department of Surgery, University of Verona Hospital Trust, Policlinico GB Rossi, Verona, Italy

^4^ Department of Surgery, University of Verona Hospital Trust, University of Verona, Verona, Italy

^5^ Pancreatic and Transplantation Surgical Unit, IRCCS San Raffaele Hospital, Vita-Salute University, Milano, Italy

^6^ Division of General and Transplant Surgery, University of Pisa, Pisa, Italy

^7^ Department of Biomedical Sciences, Humanitas University, Pieve Emanuele, Milan.

^8^ IRCCS Humanitas Research Hospital, Pancreatic Surgery Unit, Rozzano, Milan

^9^ Department of Medical and Surgical Sciences, Fondazione Policlinico Universitario A. Gemelli IRCCS, Rome, Italy

^10^ Dipartimento di Medicina e Chirurgia Traslazionale, Università Cattolica del Sacro Cuore, Roma, Italy

^11^ Unit of HPB Surgery, Pederzoli Hospital, Peschiera del Garda, Verona, Italy

^12^ Unit of Pancreatic Surgery, Department of Engineering for Innovation Medicine, Pancreas Institute, University of Verona, Verona, Italy

**Corresponding author**

During the editorial process: Prof. Salvatore Paiella, Pancreatic Surgery Unit, Department of Surgery, Dentistry, Paediatrics and Gynaecology, University of Verona, Policlinico GB Rossi, Piazzale Scuro 10, 37134, Verona, Italy; tel +390458126009; Salvatore.paiella@univr.it; post-acceptance: Prof. Ugo Boggi, Division of General and Transplant Surgery, University of Pisa, Cisanello Hospital, Via Paradisa, 2, Pisa, Italy.

Name and address **ORCID ID Salvatore Paiella 0000-0001-6893-8618**; **Twitter** @totuccio1983

**Supplementary Materials - Index**

| **Supplementary Methods** |  |
| --- | --- |
| Interaction term formula | *pag. 3* |
| **Supplementary Figures and Tables** |  |
| Table S1. Preoperative complexity analysis between LLP and RLP according to mCRS  Table S2. Univariate analysis of risk factors for conversion and unadjusted OR  Table S3. Sensitivity analysis of operative time thresholds  Figure S1. Temporal trends in minimally invasive left pancreatectomy: volume and conversion rates  Figure S2. Conversion rate according to preoperative risk  Figure S3. Receiver operating characteristic (ROC) curve – modified Conversion Risk Score (mCRS)  Figure S4. Impact of surgical approach on conversion risk by patient complexity  Figure S5. Forest plot of center-specific conversion rates with Wilson confidence interval.  Figure S6. Funnel plot of center-specific conversion rates. | *pag. 4*  *pag. 4*  *pag 5*  *pag. 6*    *pag 7*  *pag. 8*  *pag. 9*  *pag. 10*  *pag. 11* |
|  |  |
|  |  |

**Supplementary Methods**

*Interaction term formula*

To evaluate whether the surgical approach (Robotic vs. Laparoscopic) modified the impact of the preoperative risk score on conversion, we utilized a logistic regression model with an interaction term. The interaction model formula was defined as follows:

$$\ln\left( \frac{p}{1-p} \right)=\beta0+\beta1 \left( mCRS \right)+ \beta2 \left( Surgical approach \right)+ \beta3 (mCRS*surgical approach)$$

where *p* represents the probability of conversion, $\beta1$is the effect of the mCRS, $\beta2$ the effect of the surgical approach, and $\beta3 the interaction effect.$A significant p-value for $\beta3$ was considered evidence of a mitigating effect of the robotic platform on the mCRS-predicted risk.

**Supplementary Figures and Tables**

**Table S1.** Preoperative complexity analysis between LLP and RLP according to mCRS

| **Variable** | **LLP**  **(n = 1,235)** | **RLP**  **(n = 892)** | **p-value** |
| --- | --- | --- | --- |
| **mCRS^*^** | 2 (1–4) | 2 (1–3) | **0.043^#^** |
| **mCRS 0–1** | 410 (33.2) | 324 (36.3%) | **<0.001^#^** |
| **mCRS 2–3** | 499 (40.4) | 395 (44.3%) |  |
| **mCRS 4–5** | 326 (26.4) | 173 (19.4%) |  |

Values in parentheses are percentages; **^*^**median with interquartile range.

LLP: laparoscopic left pancreatectomy; RLP: robot-assisted left pancreatectomy; mCRS: modified Conversion Risk Score

^#^ Mann–Whitney U test

^#^χ² test for mCRS categories

**Table S2.** Univariate analysis of risk factors for conversion and unadjusted OR

| **Variable** | **Unadjusted OR (95% CI)** | **p-value^*^** |
| --- | --- | --- |
| **Male gender** | 1.45 (1.06-1.96) | **0.019** |
| **BMI ≥25 kg/m²** | 1.40 (1.03-1.91) | **0.035** |
| **Age ≥61** | 3.01 (1.75-5.18) | **<0.001** |
| **Pancreatic malignancy** | 3.23 (2.37-4.41) | **<0.001** |
| **Prior abdominal open surgery** | 1.36 (0.99-1.89) | 0.068 |

BMI: body mass index

^Values in bold are statistically significant^

**Table S3.** Sensitivity analysis of operative time thresholds

| **Operative time**  **threshold**  **(min)** | **Cases**  **(n)** | **Conversions**  **(n)** | **Conversion rate**  **(%)** |
| --- | --- | --- | --- |
| ≥240 | 1,246 | 138 | 11.1% |
| ≥300 | 655 | 86 | 13.1% |
| ≥360 | 335 | 49 | 14.6% |

**Figure S1.** Temporal trends in minimally invasive left pancreatectomy: volume and conversion rates

Bar plot showing annual surgical volume (left y-axis) and line plot showing conversion rate to open surgery (right y-axis) from 2019 to 2023. Although a gradual numerical decrease in conversion rates is observed over time, the Cochran–Armitage test did not demonstrate a statistically significant temporal trend.

**Figure S2.** Receiver operating characteristic (ROC) curve – modified Conversion Risk Score (mCRS)

**Figure S3.** Impact of surgical approach on conversion risk by patient complexity

Observed conversion rates according to the modified Conversion Risk Score (mCRS) category, stratified by surgical approach (Laparoscopic vs. Robot-assisted). The shaded area represents the absolute reduction in conversion risk associated with the robotic approach across increasing levels of patient complexity.

**Figure S4**. Forest plot of center-specific conversion rates with Wilson confidence interval.

Forest plot showing conversion rates for all participating centers, expressed as percentages with corresponding 95% Wilson confidence intervals. Centers are anonymized and identified by center ID, with the number of included procedures reported for each center in brackets. The wide confidence intervals observed in low-volume centers reflect small sample sizes.

**Figure S5**. Funnel plot of center-specific conversion rates.

Funnel plot displaying crude conversion rates as a function of center volume. Each dot corresponds to a center. Dashed and dotted lines indicate 95% and 99.8% control limits, respectively, illustrating the contribution of case volume to apparent inter-center variability. This representation highlights the wide dispersion of conversion rates among low-volume centers. It confirms that centers with higher volumes cluster around the overall mean, supporting the absence of a substantial independent center effect.

**Figure S6.** Risk-adjusted funnel plot of conversion by center.

Observed-to-expected conversion ratios by center, adjusted for patient-level risk factors (age ≥61 years, tumor diameter ≥35 mm, and preoperative suspicion of pancreatic malignancy). Dashed and dotted lines represent 95% and 99.8% control limits, respectively.
